# Understanding the Clinical Characteristics and Timeliness of Diagnosis for Patients Diagnosed With Long Covid: A Retrospective Observational Cohort Study From North West London

**DOI:** 10.1111/hex.70429

**Published:** 2025-09-25

**Authors:** Denys Prociuk, Jonathan Clarke, Nikki Smith, Ruairidh Milne, Cassie Lee, Simon de Lusignan, Ghazala Mir, Johannes De Kock, Erik Mayer, Brendan C. Delaney, LOCOMOTION Consortium

**Affiliations:** ^1^ I‐X, Imperial College London London UK; ^2^ Department of Mathematics Centre for Mathematics of Precision Healthcare, Imperial College London London UK; ^3^ Person with Long Covid, Member of the LOCOMOTION Study Patient Advisory Group Windsor UK; ^4^ Person with Long Covid; and Emeritus Professor of Public Health University of Southampton Southampton UK; ^5^ Imperial College London NHS Trust, Imperial College Respiratory Research Unit (ICRRU) London UK; ^6^ Nuffield Department of Primary Care Health Sciences University of Oxford Oxford UK; ^7^ Leeds Institute of Health Sciences University of Leeds Leeds UK; ^8^ NHS Highland, COVID Recovery Service Inverness UK; ^9^ iCARE Digital Collaboration Space & Secure Data Environment, NIHR Imperial Biomedical Research Centre, Imperial College Healthcare NHS Trust & Faculty of Medicine, Imperial College London London UK

## Abstract

**Background:**

Long Covid is a multisystem condition first identified in the Covid‐19 pandemic, characterised by a wide range of symptoms including fatigue, breathlessness and cognitive impairment. Considerable disagreement exists in who is most at risk of developing long Covid, driven in part by incomplete coding of a long Covid diagnosis in medical records.

**Objective:**

To describe the incidence and impact of long Covid.

**Design:**

A retrospective observational cohort study.

**Setting and Participants:**

An integrated primary and secondary care dataset from North West London, covering over 2.7 million patients. Patients with long Covid were identified through clinical terms in their primary care records.

**Main Variables Studied:**

Multivariate logistic regression was used to identify factors associated with having a long Covid diagnosis, while multivariate quantile regression was used to identify factors predicting the time a long Covid diagnosis was recorded.

**Results:**

A total of 6078 patients were identified with a long Covid clinical term in their primary care record, 0.33% of the total registered adult population. Women, those aged 41–70 years or of Asian or mixed ethnicity, were more likely to have a recorded long Covid diagnosis, alongside those with pre‐existing anxiety, asthma, depressive disorder or eczema and those living outside of the least or most socio‐economically deprived areas. Men, those aged 41–70 years, or of black ethnicity, were diagnosed earlier in the pandemic, while those with depressive disorder were diagnosed later.

**Discussion:**

Long Covid is poorly coded in primary care records, and significant differences exist between patient groups in the likelihood of receiving a long Covid diagnosis. A recorded long Covid diagnosis is more likely in women, some ethnic minority patients and those with pre‐existing long‐term conditions.

**Conclusion:**

The experience of patients with long Covid provides a crucial insight into inequities in access to timely care for complex multisystem conditions and the importance of effective health informatics practices to provide robust, timely analytical support for front line clinical services.

**Patient and Public Contribution:**

This study was co‐designed, conducted and written in conjunction with people with long Covid.

## 1 Introduction

Five years since the start of the coronavirus (Covid‐19) pandemic, health systems are still uncertain on how and where best to diagnose and treat long Covid, a complex multisystem condition resulting from acute Covid‐19 infection [1]. Current evidence suggests long Covid may represent a diverse set of pathophysiological processes, including viral persistence or reactivation, autoimmune responses, end‐organ damage and autonomic dysfunction [2, 3]. This in turn may produce a wide range of symptoms and clinical sequelae for patients, influenced by the particular way in which long Covid has manifested and how it interacts with an individual's pre‐existing conditions [1, 4, 5, 6, 7].

As a new condition without established clinical pathways, patients may experience challenges in navigating the healthcare system to obtain an appropriate and timely diagnosis. This is particularly the case for long Covid, where its symptom profile is particularly broad, encompassing a diverse range of symptoms including fatigue, breathlessness, cognitive dysfunction and palpitations [1, 4].

There is evidence that electronic health records under‐record the prevalence of long Covid. Self‐reported prevalence of long Covid has been estimated at 3.3% in England and Scotland by the Office for National Statistics, between 6.6% and 10.4% in a national survey in Scotland and 7% in a large survey from the United States [8, 9, 10]. Conversely, studies examining the presence of clinical codes for long Covid in patient records estimate a prevalence of 0.02% [11]. Others have found that of those with self‐reported long Covid, only 5.4% had a recorded diagnosis in their medical record, rising to 6.3% for those with severe symptoms, suggesting significant under‐coding or under‐diagnosis of long Covid [12].

Several studies have identified factors associated with a long Covid diagnosis. Across studies, women and those in middle and older age are more likely to self‐report long Covid symptoms and receive a long Covid diagnosis [8, 9, 13, 14]. Similarly, vaccination against Covid‐19 has been associated with reduced risk of long Covid, while a diagnosis of one or more of several long‐term conditions, including asthma, chronic obstructive pulmonary disease (COPD), anxiety, depression and type 2 diabetes (T2D), has been associated with higher risk of developing long Covid [8, 9, 13, 14]. Importantly, the relationship between long Covid and long‐term conditions may also differ according to patient demographic characteristics. A study exploring the association between long Covid and T2D found long Covid to be more common in men with T2D than matched controls, but the reverse for women [15].

Considering ethnicity, white respondents were more likely to self‐report long Covid symptoms than black respondents in a US survey and more likely to have a recorded long Covid diagnosis. In a UK study, however, Asian participants in the ONS Covid Infection Survey were more likely to report long Covid symptoms (4.1%) than white (3.3%) and black participants (1.8%) [8, 10, 14]. In terms of socio‐economic position, adults living in regions in the most socio‐economically deprived quintile of England and Scotland were most likely to self‐report long Covid symptoms (5.6%) than those in the least deprived quintile (2.8%), but are less likely to have a recorded long Covid diagnosis [10, 14, 16].

Although showing some agreement, these studies highlight important differences between self‐reported and diagnosed long Covid between patient groups. This may indicate greater barriers to receiving a long Covid diagnosis for some patients than others. To examine this further, this study uses an integrated electronic patient record dataset to examine the scale of long Covid diagnosis in the population of North West London. We aim to identify factors that may be predictive of receiving a long Covid diagnosis, identify factors that predict the time of long Covid diagnosis, and quantify the prevalence of new long‐term conditions amongst patients with long Covid.

## 2 Methods

### 2.1 Data Sources

The Whole Systems Integrated Care database (WSIC), hosted in the iCARE Secure Data Environment, was the primary source of data used for this study. The WSIC database contains record‐level data derived from primary care records of over 2.8 million patients registered to 350 GP practices in North West London and linked to hospital Secondary Uses Service data containing records of appointments, Emergency Department (ED) attendances and inpatient admissions.

### 2.2 Data Analysis

Patients were identified as having long Covid if their primary care record contained one of four long Covid Systematized Nomenclature of Medicine (SNOMED) clinical terms after 1 January 2020. These codes were chosen based on their suggested use in clinical coding by NHS England upon their creation as SNOMED codes in January 2021 [17]. Other studies have used two UK‐specific SNOMED clinical terms for long Covid, specifically 1325161000000102 (Post‐Covid‐19 syndrome) and 1325181000000106 (Ongoing symptomatic Covid‐19); however, our study also includes international SNOMED codes to account for variation in coding norms between general practitioners, specifically 1119303003 (Post‐acute Covid‐19) and 1119304009 (Chronic post‐Covid‐19 syndrome) [17, 18].

The presence of diagnostic clinical terms for each of 20 long‐term conditions (listed in Table 1) was identified using lists of SNOMED codes assigned to each condition obtained from the Oxford Royal College of General Practitioners Clinical Informatics Digital Hub (ORCHID) database (see Supporting Information for code lists) [19]. The date each condition first appeared in a patient's clinical record was also noted.

**Table 1:** Demographic and clinical characteristics of patients with a recorded long Covid diagnosis and the overall adult WSIC patient population.

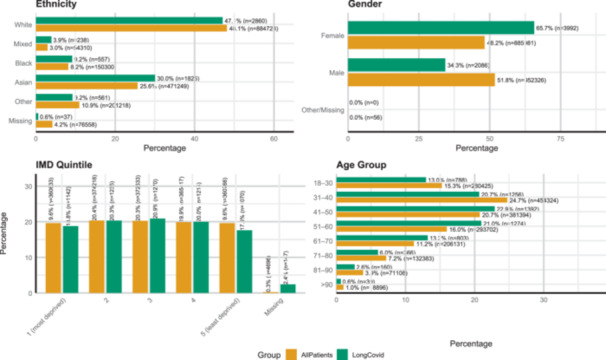

The age, sex and ethnicity of the long Covid population were described and compared to patients without a diagnosis of long Covid, along with the distribution of Index of Multiple Deprivation ranks (a measure of socio‐economic status) of the Lower Layer Super Output Area of residence of those with and without a diagnosis of long Covid. The prevalence of the 22 long‐term conditions in the long Covid patient population from 1 January 2017 to 31 December 2019 was compared to those without long Covid in the WSIC dataset. Multivariate logistic regression, including the panel of demographic covariates and comorbidities, was used to identify factors predictive of receiving a diagnosis of long Covid. Age was transformed into deciles, with the exception of those aged 18–30 who were grouped together, and treated as a categorical variable with those aged 18–30 as the reference group.

The incidence of new clinical comorbidities in the period from 1 January 2020 to 5 December 2023 in the long Covid population was described and compared to those without a recorded long Covid diagnosis. 1 January 2020 was chosen in this case to identify all additional clinical comorbidities acquired since the start of the pandemic. As a supplementary analysis, *K*‐means clustering was used to group patients with a long Covid diagnosis according to the clinical comorbidities they had been diagnosed with before 1 January 2020 and separately the new conditions acquired on or after 1 January 2020. Optimal configurations were determined using the elbow method. The relationship between these two configurations was compared using a Sankey diagram.

For those patients with a recorded long Covid diagnosis, the time between 1 January 2020 and the date of long Covid diagnosis was calculated. Multivariate median regression was used to identify factors predictive of patients receiving a long Covid diagnosis later than others. Age was recoded as described above.

### 2.3 Software

Data extraction was performed using Microsoft SQL Server Management Studio 2018; data processing and analysis was conducted using Python version 3.7.9 and the Pandas version 1.3.2 and numpy version 1.19.5 libraries. Statistical analysis was performed using Stata version 15.

## 3 Results

**Figure 1:**
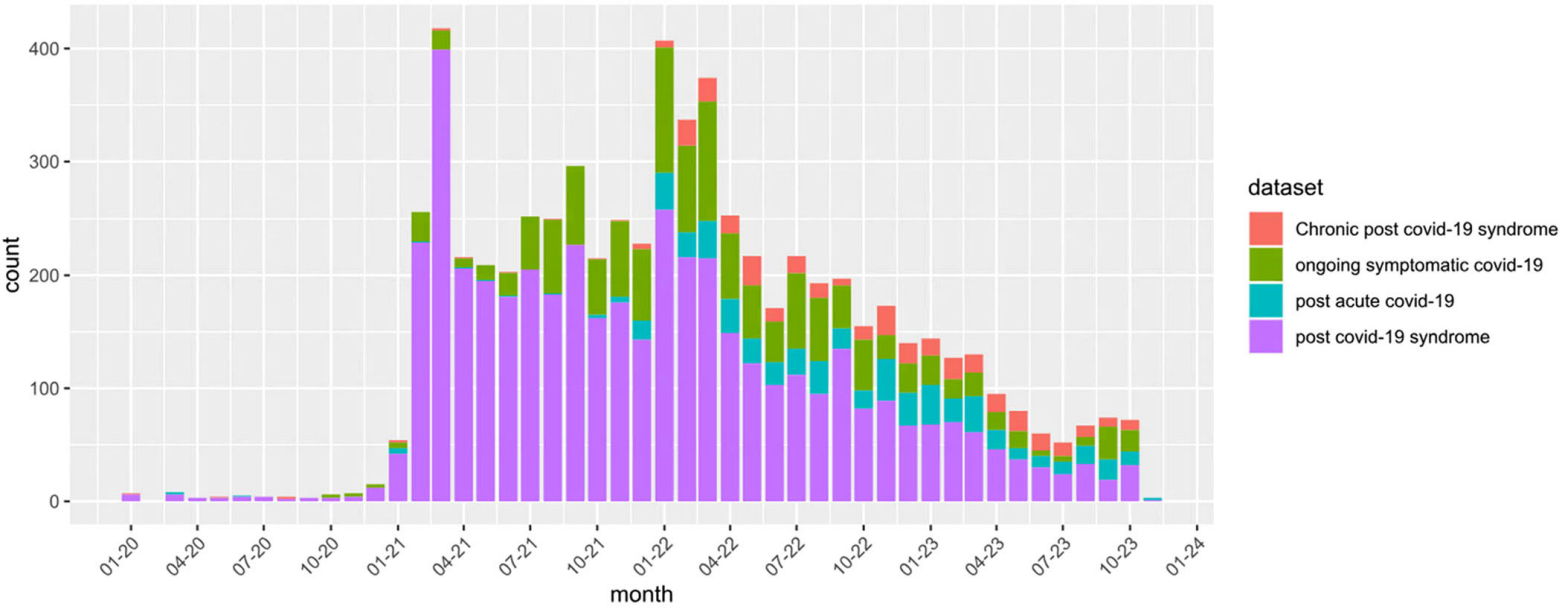
Frequency of new long Covid diagnoses, disaggregated by the diagnosis code used.

The WSIC dataset contained clinical records for 1,838,363 adult patients registered to General Practices within Northwest London as of 5 December 2023. Of these patients, 6078 (0.33%) were identified as having a long Covid diagnosis code. Figure 1 shows the number of new diagnoses of long Covid recorded each month since 1 January 2020. A peak in diagnosis frequency is seen in the first months of 2021, corresponding to the introduction of long Covid SNOMED diagnosis codes. Over time, a gradual decline in diagnosis frequency is seen, alongside a change in coding practices. The initially dominant ‘Post‐COVID‐19 syndrome’ (1325161000000102) code is increasingly superseded by the other three codes, creating a mixed coding picture in the most recent diagnoses.

Table 1 shows the demographic characteristics of the long Covid population compared with the overall population of adult patients registered to GP practices within North West London. Patients with a long Covid diagnosis were statistically significantly older (mean age 48.7 years vs. 48.0 years, *p* < 0.0001) and more likely to be female (65.7% vs. 48.2%, *p* < 0.0001) than those without a long Covid diagnosis. Patients recorded as Asian or Asian British ethnicity accounted for 30.03% of long Covid patients compared to 25.63% the overall population. Patients with missing ethnicity data represented 0.61% of the long Covid population, but 4.16% of the overall WSIC population. Patients living in areas in the most socio‐economically deprived quintile of North West London accounted for 19.25% of those with a long Covid diagnosis and 18.79% of those without a diagnosis, while patients living in the least deprived quintile of areas accounted for 17.60% of patients with a long Covid diagnosis and 19.62% of patients those without.

As shown in Table 2, before the Covid‐19 pandemic, 2574 (42.3%) patients who went on to have a long COvid diagnosis had one or more of the 20 included clinical comorbidities, compared to 3504 patients who did not. Compared with the overall population, asthma (12.92% vs. 3.78%), anxiety (12.37% vs. 3.30%), depressive disorder (10.99% vs. 3.31%), hypertension (10.64% vs. 4.73%) and type 2 diabetes mellitus (7.70% vs. 3.35%) were more common in those with a recorded long Covid diagnosis.

**Table 2:** Number of patients with a recorded diagnosis of each included clinical comorbidity in the period from January 2017 to 31 December 2019, compared to new conditions recorded after 1 January 2020, excluding those with a pre‐existing diagnosis of that condition.

	2017–2019					2020–2023				
	Long Covid patients		All patients		*χ* ^2^	Long Covid patients		All patients		*χ* ^2^
Condition	*N*	%	*N*	%	*p*	*N*	%	*N*	%	*p*
Anxiety	752	12.37	84,049	4.57	< 0.001	982	18.44	71,210	4.06	< 0.001
Asplenia	*	*	272	0.01	*	*	*	296	0.02	*
Asthma	785	12.92	1,06,093	5.77	< 0.001	378	7.14	27,443	1.58	< 0.001
Atrial fibrillation	67	1.10	24,266	1.32	0.1364	93	1.55	25,066	1.38	0.2711
Chronic kidney disease	91	1.50	20,120	1.09	0.0025	177	2.96	31,082	1.71	< 0.001
Chronic liver disease	5	0.08	2372	0.13	0.309	15	0.25	2369	0.13	0.0103
Chronic lung disease	75	1.23	17,753	0.97	0.0322	91	1.52	8757	0.48	< 0.001
Cirrhosis of the liver	*	*	1391	0.08	*	10	*	1533	0.08	*
COPD	78	1.28	17,834	0.97	0.0126	75	1.25	8118	0.45	< 0.001
Dementia	22	0.36	8846	0.48	0.1785	45	0.74	8981	0.49	0.0049
Depressive disorder	668	10.99	87,399	4.75	< 0.001	811	14.99	62,020	3.54	< 0.001
Diabetes type 1	23	0.38	5510	0.30	0.261	15	0.25	1657	0.09	< 0.001
Diabetes type 2	468	7.70	1,05,875	5.76	< 0.001	261	4.65	39,766	2.30	< 0.001
Eczema	286	4.71	44,285	2.41	< 0.001	256	4.42	30,235	1.69	< 0.001
Haemorrhagic stroke	5	0.08	1636	0.09	0.8601	10	0.16	2284	0.12	0.3719
Heart failure	30	0.49	10,804	0.59	0.3363	84	1.39	15,543	0.85	< 0.001
Hypertension	647	10.64	1,50,350	8.18	< 0.001	547	10.07	99,731	5.91	< 0.001
Ischaemic heart disease	77	1.27	19,182	1.04	0.086	130	2.17	18,628	1.02	< 0.001
Ischaemic stroke	*	*	1829	0.10	*	16	*	2788	0.15	*
Peripheral vascular disease	*	*	970	0.05	*	*	*	1502	0.08	*

*Denotes values below 5.

### 3.1 Factors Predictive of Receiving a Long Covid Diagnosis

**Figure 2:**
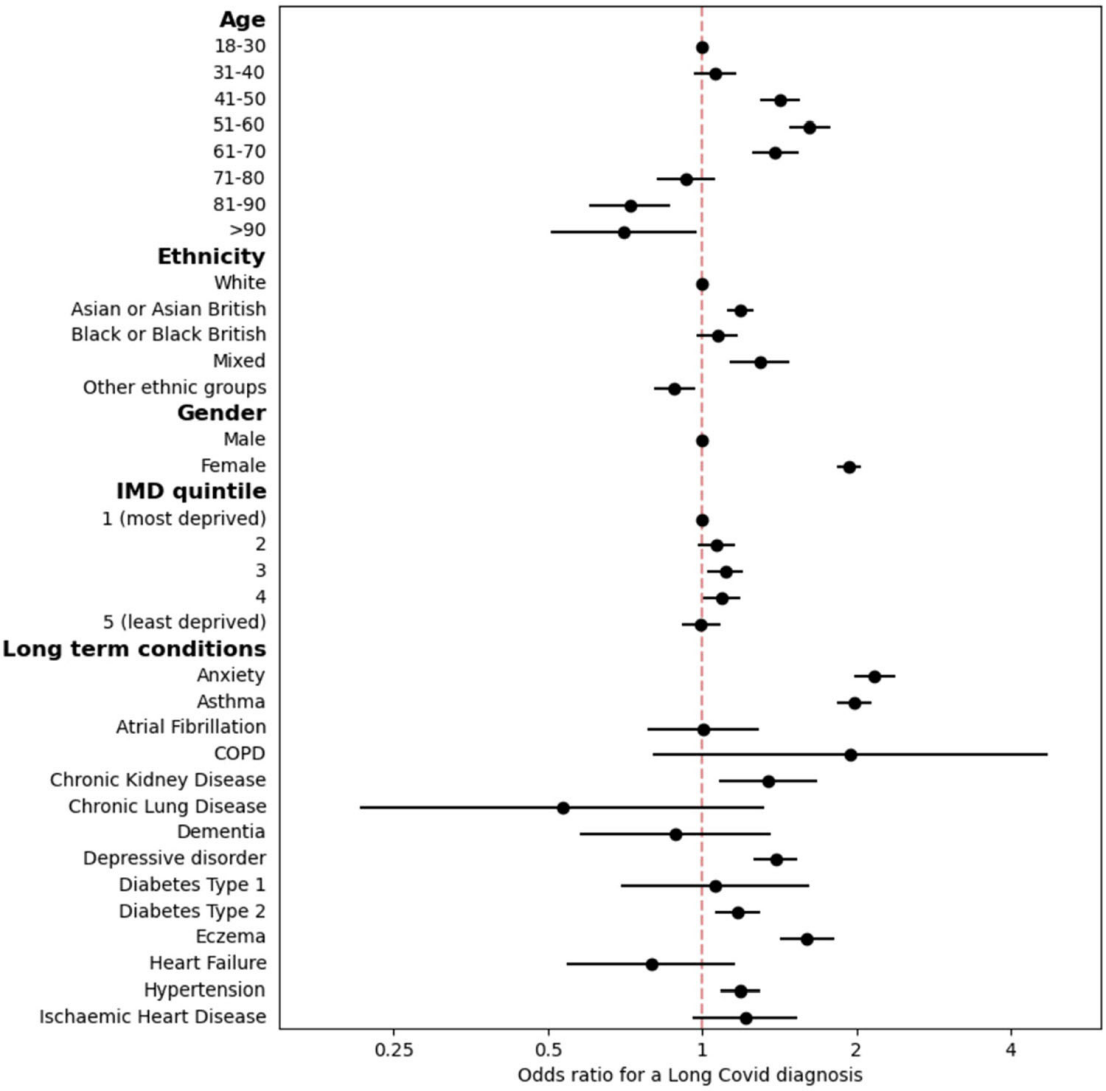
Adjusted odds ratios for having a recorded long Covid diagnosis code in the primary care record. Comorbidities relate to conditions diagnosed before 1 January 2020. Bars show 95% confidence intervals of the odds ratio.

The above analyses reflect the univariate relationships between the clinical and demographic features of patients and their likelihood of receiving a long Covid diagnosis. To account for the correlation between these variables, multivariate logistic regression was performed to estimate the odds ratio for having a recorded long Covid diagnosis, accounting for a patient's demographic and clinical characteristics before 1 January 2020. Figure 2 shows the adjusted odds ratios for the multivariate logistic regression model for diagnosis of long Covid.

Compared to the reference group of ages 18–30, those aged 31–70 years were significantly more likely to receive a long Covid diagnosis, peaking at 51–60 years (aOR 1.62, *p* < 0.001). Those aged over 80 years were significantly less likely to receive a long Covid diagnosis than those aged 18–30 years. Patients of Asian or Asian British or mixed ethnicity were significantly more likely than patients of White ethnicity to receive a long Covid diagnosis (aOR 1.19 and 1.29, *p* < 0.001). Patients whose ethnicity was recorded as ‘Other Ethnic Group’ had significantly lower odds of receiving a long Covid diagnosis (aOR 0.88, *p* = 0.009).

Women were significantly more likely to have received a long Covid diagnosis than men (aOR 1.93, *p* < 0.001). Those living in the least socio‐economically deprived quintile of areas were no more or less likely to have a recorded long Covid diagnosis than those in the most deprived quintile. Those living in the third and fourth most deprived quintiles were significantly more likely to have a recorded long Covid diagnosis than those in the most deprived quintile (aOR 1.11, *p* = 0.011 and 1.19, *p* = 0.035). Patients with recorded diagnoses of several long‐term conditions before the Covid‐19 pandemic were significantly more likely to receive a long Covid diagnosis, specifically anxiety (aOR 2.17), asthma (aOR 1.98), eczema (aOR 1.59), chronic kidney disease (aOR 1.34), depressive disorder (aOR 1.39), hypertension (aOR 1.19) and T2D (aOR 1.17). No conditions were associated with significantly lower odds of diagnosis of long Covid; however, small sample sizes for some less common conditions lead to uncertain estimates.

### 3.2 Factors Predictive of an Earlier Long Covid Diagnosis

**Figure 3:**
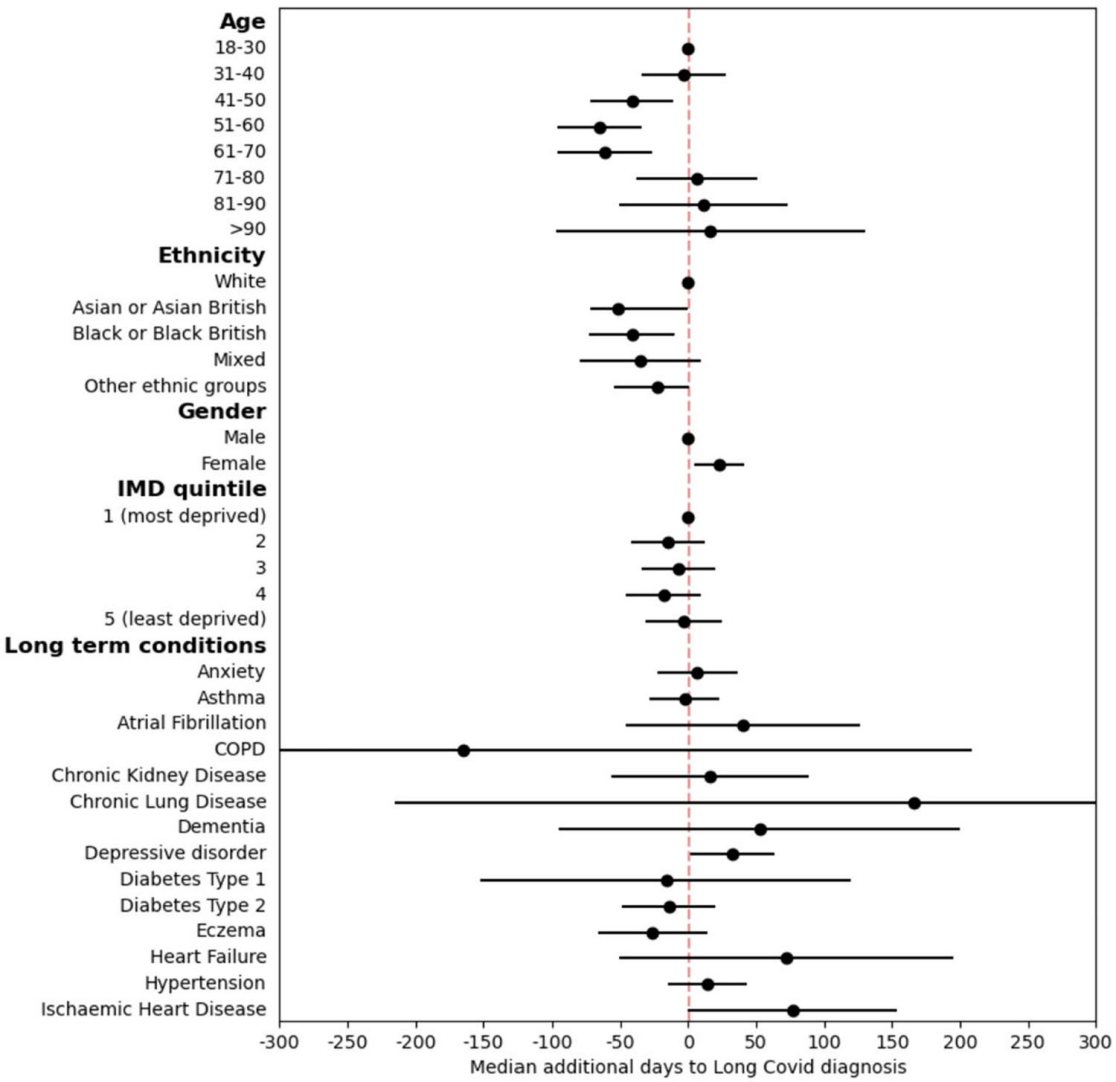
Predicted difference in median time to long Covid diagnosis, obtained from a multivariate median quantile regression model. Comorbidities relate to conditions diagnosed before 1 January 2020.

Figure 3 shows the results of a multivariate median regression model estimating the time from 1 January 2020 to the date of long Covid diagnosis for patients with a recorded long Covid diagnosis. Compared to those aged 18–30 years, patients aged 41–70 years were diagnosed with long Covid significantly earlier in the pandemic, with those aged 51–60 years estimated to be diagnosed an average of 62.9 days earlier than those aged 18–30. Compared to White patients, people recorded as Asian (−52.0 days, *p* < 0.001) or black ethnicity (−41.3 days, *p* = 0.01) were diagnosed significantly earlier in the pandemic. Women waited significantly longer than men (22.7 days, *p* = 0.015). No significant differences with respect to socio‐economic status were observed. Across the 20 conditions, only depressive disorder (32.7 days, *p* = 0.040) was associated with a statistically significant difference in the time of diagnoses, with large uncertainty in the estimates of less common conditions.

### 3.3 Acquisition of New Conditions Following a Long Covid Diagnosis

Around 40% of patients (2574, 42.3%) with a diagnosis of long Covid also had another new clinical condition entered into their primary care record in the period since 1 January 2020. As shown in Table 2, of those patients without a previous diagnosis of anxiety, 982 (18.44%) have received a new diagnosis of anxiety since 1 January 2020, compared to 4.06% of all patients. Similarly, of those without a previous diagnosis of depressive disorder, 811 (14.99%) have received a diagnosis of depressive disorder, compared to 3.54% overall. Other chronic conditions have also been newly diagnosed often, including hypertension (547 patients, 10.07% vs. 5.91% overall), asthma (378 patients, 7.14% vs. 1.58% overall) and type 2 diabetes mellitus (261 patients, 4.65% vs. 2.30% overall). As shown in the supplementary results, patients who go on to receive a long Covid diagnosis may be grouped into distinct clusters, largely centred around common conditions such as asthma, hypertension or T2D (Figure S2). Tracking these patients after the onset of the Covid‐19 pandemic, we see a wide range of clinical trajectories in the development of new clinical comorbidities, with no dominant trajectories for patients based on their pre‐existing conditions (Figure S5).

## 4 Discussion

### 4.1 Summary of Findings

In this study of primary care record data from North West London, we find far lower prevalence of recorded long Covid diagnoses (0.33%) than expected based on self‐reported survey data from the UK Office for National Statistics (3.3%) and from a nationwide Scottish survey (6.6%–10.3%), suggesting significant undercoding in primary care [9, 10]. This is supported by recent linkage of self‐reported long Covid to primary care records, which found that as few as 5.4% of patients with self‐reported long Covid had a recorded long Covid diagnosis [12]. Using UK‐specific and international SNOMED clinical concepts for long Covid, we see changes in coding practice over time, supporting the use of this wider set of codes in future analyses. Indeed, our long Covid prevalence of 0.33% using diagnosis codes alone is far higher than the 0.02% prevalence observed using a similar method in Scotland [11]. It is likely that the extent of underdiagnosis of long Covid varies between patient groups. Recent research from the United Kingdom has identified barriers in access to long Covid care for patients from socially excluded populations, including a lack of awareness of long Covid and a mistrust of healthcare services based on previous personal or community experience of healthcare discrimination [20].

Our finding of a recorded long Covid diagnosis being more common in women and in those of middle age is consistently observed across other studies [9, 11, 14]. We find that those of Asian or mixed ethnicity were significantly more likely to receive a long Covid diagnosis than white patients, a finding not observed in some studies, and the reverse was identified in others [9, 14, 21]. As in other studies, we find a trend towards a higher risk of recorded long Covid in those who do not live in the most socio‐economically deprived areas; however, we do not find that this association extends to the least deprived quintile of our sample [9, 14]. This aligns with a lower prevalence of long Covid in the least deprived quintile of Scottish residents [8, 11]. As those living in the most socio‐economically deprived areas of the United Kingdom are known to have a higher risk of Covid‐19 infection than those in the least deprived areas, finding similarities in the odds of having a recorded long Covid diagnosis between the most and least deprived areas in our study is important. Lower recorded long Covid diagnosis in the most deprived areas may indicate underdiagnosis, potentially through barriers to access to care, as reported elsewhere [20]. The lower risk of Covid‐19 infection in those living in the least deprived areas helps explain the low rate of long Covid diagnosis found in these areas. In addition, the use of private healthcare services may play a role in differences in long Covid prevalence with respect to deprivation. Patients able to afford private healthcare may have sought management of their long Covid symptoms there [22], and diagnoses may not have been added to the patient's NHS primary care record, contributing further to the low rate of long Covid diagnosis in the least deprived areas.

Around four in ten patients (42.3%) who went on to receive a long Covid diagnosis had a pre‐existing long‐term condition, with pre‐existing asthma, eczema, anxiety and depressive disorder being strongly associated with a long Covid diagnosis. Conversely, six in ten patients who received a long Covid diagnosis had not been diagnosed with any of the twenty included clinical comorbidities before the pandemic. Since the onset of the Covid‐19 pandemic, however, patients who received a long Covid diagnosis have experienced a significant increase in the burden of mental health conditions, particularly with 18.4% of those without a previous diagnosis of anxiety going on to receive a new diagnosis of anxiety since 2020, compared to 4.0% of the overall population. Although it is not possible to infer a causal link, similar patterns are seen for depressive disorder, suggesting an important role for mental health services. Increased new diagnoses of hypertension, asthma and diabetes are observed, but again, potential detection of common pre‐existing conditions at a long Covid‐related consultation is quite likely. As the supplementary results indicate, patients with long Covid have varied clinical trajectories in the new conditions they develop, rather than following a small set of patterns of disease.

An earlier diagnosis of long Covid in the clinical record may reflect either earlier onset of long Covid, a more timely recording of the diagnosis or a combination of the two. Thus, those exposed to Covid‐19 earlier in the pandemic may be more likely to develop long Covid earlier and therefore receive a recorded diagnosis earlier. We find middle‐aged patients, those recorded as Asian or mixed ethnicity, and men were more likely to be diagnosed with long Covid earlier in the pandemic. This finding may partly be influenced by the disproportionate number of black and Asian workers in key workforce sectors in London [23]. Although 58% of key workers are female, large differences exist across occupations, with 90% of key workers in transport being male, while 79% of those in health and social care were female [24]. Research on the timeliness of long Covid diagnoses is limited; however, factors related to underdiagnosis of long Covid, reflecting barriers to accessing healthcare, patient awareness of long Covid and health‐seeking behaviours are also likely to influence the timeliness of diagnoses where one is made. Our finding that middle‐aged adults are generally diagnosed earliest may reflect the need to promptly link long Covid symptoms to a clinical diagnosis as a result of their impact on employment and the need to receive clinical recognition of their condition for their employers [25]. Clinical comorbidities do not strongly influence the time of diagnosis, except for depression which was associated with diagnosis an average of 35 days later, suggesting potential barriers to receiving a timely diagnosis for those with poor mental health.

### 4.2 Strengths and Limitations

The study uses SNOMED codes from both the United Kingdom and International code lists to account for variation on long Covid coding between practitioners. Using this strategy, we identify an additional 729 patients that would not otherwise have been included using UK codes alone. We also include codes for both long Covid and post‐Covid conditions to identify patients with persistent symptoms after a Covid‐19 infection.

Despite this strategy, it is likely that we have not identified a significant number of patients with long Covid, but for whom a relevant clinical term does not appear in their primary care record. This may result from diagnoses being made outside of primary care and not reported back to and/or coded by their general practitioner, patients who have not received a formal long Covid diagnosis or where a code for another condition has been used. Other studies have used a symptom‐based approach to identify patients whose clinical symptoms, as recorded in the primary care record, are consistent with long Covid [6, 21]. While it was not possible in this study to apply these methods, it is expected that using this approach would identify new cases of likely long Covid and also identify patients with a recorded long Covid diagnosis whose recorded symptoms may not align with a recognised long Covid phenotype. Similarly, self‐reported survey data on long Covid symptoms are known to identify large numbers of patients who may have long Covid but do not have a recorded diagnosis in their medical record [12, 15]. It was not possible to use self‐reported data in this study, and doing so would introduce the challenge of distinguishing those who perceived they had long Covid from those who met clinical diagnostic criteria for the condition, introducing its own biases.

We chose to focus on the relationship between long Covid and a set of 20 common, relevant long‐term conditions. These conditions represent a small subset of all long‐term conditions, and it may be that associations with other conditions may emerge should a larger set be used. As this study includes only patients registered to GP practices in North West London, the findings of the study may not be generalisable to other regions. The region is entirely urban, and younger and more ethnically diverse than England as a whole.

In this study, we were unable to precisely determine the date of Covid‐19 infection and the date of onset of long Covid symptoms. Instead, 1 January 2020 was chosen over the date of a positive Covid‐19 test as the index date for the time to diagnosis and to distinguish between pre‐ and post‐pandemic clinic activity, for three reasons. Firstly, many patients within the dataset had multiple positive Covid‐19 tests, separated over time and occurring both before and after their long Covid diagnosis date. As long Covid may result from the first or a subsequent Covid‐19 infection, it is not possible to determine which positive Covid‐19 test should be chosen. Secondly, many patients did not have a positive Covid‐19 test in their clinical record. In these cases, using a Covid‐19 test date to determine time to diagnosis would exclude these patients. Thirdly, feedback from the study's Patient Advisory Group suggested that, particularly early in the Covid‐19 pandemic, Covid‐19 tests were not widely available and a diagnosis of Covid‐19 was often based on clinical grounds alone, after which long Covid symptoms developed. It was therefore not possible to precisely quantify the time from Covid‐19 infection to the development of long Covid symptoms or the diagnosis of long Covid. Some conditions diagnosed after 1 January 2020 may predate Covid‐19 infection or the occurrence of long Covid symptoms; however, taking the date of long Covid diagnosis instead may exclude comorbidities diagnosed during the period between the onset of long Covid symptoms and its diagnosis.

### 4.3 Implications for Policy and Practice

The analysis of large health record datasets offers an important insight into how care is provided within a health system and how provision may vary between patient groups. Such studies rely on having a means to robustly identify the condition of interest, which in the case of long Covid is notably lacking. A recent study used a range of methods to attempt to identify patients with long Covid from primary care data, finding variable prevalence from 0.02% using diagnosis codes alone, 0.2% when using natural language processing to review clinical free text, 0.3% using data from sick notes and 1.7% when using a phenotype of clinical symptoms and patterns of activity [11]. Crucially, the overlap between these different means of identification was generally low, supporting the use of multiple contrasting identification strategies.

Our study has demonstrated that delays in the introduction and implementation of long Covid clinical terms precluded the timely recording of long Covid diagnoses in health records. Specific clinical terms for long Covid did not enter widespread clinical use until almost a year after the onset of the Covid‐19 pandemic in the United Kingdom. Further, disagreement between NHS and international coding recommendations appears to have produced inconsistent coding practices between clinicians and over time [5, 6, 26]. Consequently, incomplete, delayed or biased recording of a long Covid diagnosis may in turn influence the findings of observational studies.

Although not all patients self‐reporting long Covid symptoms may meet the diagnostic criteria for the condition, the finding of at least a ten‐fold difference in prevalence between the lower bound of national surveys (3.3%) and our study (0.33%) is deeply concerning. Efforts should continue to be made in primary and secondary care settings to identify and properly code patients who meet the diagnostic criteria for long Covid.

While we find agreement between factors predisposing self‐reported and diagnosed long Covid for women, those in middle age and of Asian and white ethnicity, concern exists that those in the most socio‐economically deprived areas of North West London may be more likely to have long Covid symptoms, but are not receiving a diagnosis at the same rate as those living in less deprived areas. Further work is also needed to understand these discrepancies and to address any associated unmet clinical needs.

A co‐ordinated approach to the clinical management of long Covid is required to address both its wide‐ranging symptoms and the high frequency with which patients with long Covid go on to develop other long‐term conditions, including mental health diagnoses. In particular, the continued importance of multidisciplinary clinics needs to be emphasised. Not only do these continue to offer integrated expert care for this challenging group of highly comorbid patients, but they also act as centres of excellence in ongoing research. Closer working between long Covid multidisciplinary teams and primary care remains important for raising and maintaining awareness of long Covid across the healthcare system.

While over 4 years have passed since the start of the Covid‐19 pandemic in the United Kingdom, many patients continue to live each day with long Covid. Quite how many is difficult to say, given delayed and incomplete diagnosis coding in clinical records, and much of what could have been learned about this new condition is to some extent now unknowable. Considering future pandemics, health systems must be more vigilant for the long‐term sequelae of novel pathogens and rapidly develop appropriate clinical pathways to diagnose and treat patients. This should include frameworks for rapid initial investigation, including integration of functional imaging, immunology and proteomics to characterise an in‐depth phenotype of any new prolonged post‐viral syndromes. The emphasis should not just be on rehabilitation, but on rapid discovery science leading to timely clinical trials of the type so successfully carried out in managing the acute condition. As part of this strategy, to improve diagnostic coding, clinical terms must be developed and consistently implemented in tandem with these services to ensure health record data can be used to rapidly support the effective delivery of care.

## 5 Conclusion

Long Covid occurs commonly within the UK population, yet is significantly under‐recorded in primary care records. The experience of patients with long Covid provides a crucial insight into inequities in access to timely care for complex multisystem conditions, and the importance of effective health informatics practices to support robust and timely analytical support for front‐line clinical services.

## Author Contributions

**Denys Prociuk:** conceptualization, data curation, formal analysis, writing – original draft, writing – review and editing. **Jonathan Clarke:** conceptualization, data curation, formal analysis, writing – original draft, writing – review and editing. **Nikki Smith:** conceptualization, writing – review and editing. **Ruairidh Milne:** conceptualization, writing – review and editing. **Cassie Lee:** conceptualization, writing – review and editing. **Simon de Lusignan:** conceptualization, writing – review and editing. **Ghazala Mir:** conceptualization, writing – review and editing. **Johannes De Kock:** conceptualization, writing – review and editing. **Erik Mayer:** conceptualization, writing – review and editing. **Brendan C. Delaney:** conceptualization, supervision, writing – review and editing.

## Disclosure

The views expressed are those of the authors and not necessarily those of the NHS, the NIHR or the Department of Health and Social Care.

## Ethics Statement

Ethics approval for the LOCOMOTION study was obtained from the Bradford and Leeds Research Ethics Committee on behalf of Health Research Authority and Health and Care Research Wales (reference: 21/YH/0276). The iCARE Secure Data Environment was given favourable ethics approval by the South West—Central Bristol Research Ethics Committee (reference 21/SW/0120; IRAS project ID 282093) and includes the Whole System Integrated Care (WSIC) database (West Midlands Solihull Research Ethics Committee [reference 18/WM/0323; IRAS project ID 252449]). All data used in this paper were fully anonymised before analysis.

## Conflicts of Interest

The authors declare no conflicts of interest.

## Locomotion Study Consortium

**Table:** 

First name	Last name	Role
Nawar	Bakerly	Principal Investigator
Kumaran	Balasundaram	NHS Clinical Research Fellow
Megan	Ball	NHS Clinical Research Fellow
Mauricio	Barahona	Co‐Investigator
Alexander	Casson	Co‐Investigator
Jonathan	Clarke	HEI Researcher
Karen	Cook	Patient Advisory Group Member
Rowena	Cooper	NHS Clinical Research Fellow
Vasa	Curcin	Co‐Investigator
Julie	Darbyshire	Co‐Investigator
Helen	Davies	Principal Investigator
Helen	Dawes	Co‐Investigator
Simon	de Lusignan	Co‐Investigator
Brendan	Delaney	Chief Investigator
Carlos	Echevarria	Principal Investigator
Sarah	Elkin	Principal Investigator
Ana Belen	Espinosa Gonzalez	HEI Researcher
Rachael	Evans	Principal Investigator
Sophie	Evans	Patient Advisory Group Member
Zacchaeus	Falope	Principal Investigator
Ben	Glampson	HEI Researcher
Madeline	Goodwin	Research Assistant
Trish	Greenhalgh	Co‐Investigator
Darren C	Greenwood	Co‐Investigator
Stephen	Halpin	Principal Investigator
Juliet	Harris	NHS Research Assistant
Will	Hinton	HEI Researcher
Mike	Horton	Co‐Investigator
Samantha	Jones	NHS Clinical Research Fellow
Joseph	Kwon	HEI Researcher
Cassie	Lee	NHS Clinical Research Fellow
Ashliegh	Lovett	NHS Clinical Research Fellow
Mae	Mansoubi	HEI Researcher
Victoria	Masey	NHS Clinical Research Fellow
Harsha	Master	Principal Investigator
Erik	Mayer	HEI Researcher
Bernardo	Meza‐Torres	HEI Researcher
Ruairidh	Milne	Patient Advisory Group Member
Ghazala	Mir	Co‐Investigator
Jacqui	Morris	Principal Investigator
Adam	Mosley	NHS Research Assistant
Jordan	Mullard	HEI Researcher
Daryl	O'Connor	Co‐Investigator
Rory	O'Connor	Co‐Investigator
Thomas	Osborne	Project Manager
Amy	Parkin	NHS Clinical Research Fellow
Stavros	Petrou	Co‐Investigator
Anton	Pick	Principal Investigator
Denys	Prociuk	HEI Researcher
Clare	Rayner	Patient Advisory Group Member
Amy	Rebane	Patient and Public Involvement Manager
Natalie	Rogers	Patient Advisory Group Member
Janet	Scott	Principal Investigator
Manoj	Sivan	Chief Investigator
Nikki	Smith	Patient Advisory Group Member
Adam	Smith	Statistician
Emma	Tucker	Principal Investigator
Ian	Tucker‐Bell	Patient Advisory Group Member
Paul	Williams	NHS Clinical Research Fellow
Darren	Winch	Patient Advisory Group Member
Conor	Wood	NHS Research Assistant

## Supporting information


**Figure S1:** Elbow plot for K‐means clustering of comorbidities for Long Covid patients between January 2017 and December 2019, vertical line indicates the plot chosen configuration of 6 clusters. **Figure S2:** Heatmap showing the prevalence of conditions within each of the 6 patient clusters defined based on K‐means clustering of comorbidities for Long Covid patients between January 2017 and December 2019. **Figure S3:** Elbow plot for K‐means clustering of comorbidities for Long Covid patients between January 2020 and September 2023, vertical line indicates the plot chosen configuration of 9 clusters. **Figure S4:** Heatmap showing the prevalence of conditions within each of the 9 patient clusters defined based on K‐means clustering of comorbidities for Long Covid patients between January 2020 and September 2023. **Figure S5:** Sankey diagram showing the assignment of patients to disease clusters in the pre‐pandemic and post‐pandemic periods. Colours of edges represent the pre‐pandemic cluster to which a patient was assigned. Numbers represent the total patients assigned to each cluster.

## Data Availability

Data accessed for this research study are held within the WSIC database tenancy hosted in the iCARE Secure Data Environment and were approved through the relevant data access committee (see ‘Ethics Statement’). Patient data is not available for sharing on account of privacy regulations. Codes used for the study and aggregate data outputs are available from the authors upon reasonable request.
