# Supplementary material for: Understanding the Clinical Characteristics and Timeliness of Diagnosis for Patients Diagnosed With Long Covid: A Retrospective Observational Cohort Study From North West London
Source: Health Expect. 2025 Sep 25;28(5):e70429. doi: 10.1111/hex.70429 (PMC12462644; doi:10.1111/hex.70429)
Supplement: Supplementary file 1 — Figure S1: Elbow plot for K‐means clustering of comorbidities for Long Covid patients between January 2017 and December 2019, vertical line indicates the plot chosen configuration of 6 clusters. Figure S2: Heatmap showing the prevalence of conditions within each of the 6 patient clusters defined based on K‐means clustering of comorbidities for Long Covid patients between January 2017 and December 2019. Figure S3: Elbow plot for K‐means clustering of comorbidities for Long Covid patients between January 2020 and September 2023, vertical line indicates the plot chosen configuration of 9 clusters. Figure S4: Heatmap showing the prevalence of conditions within each of the 9 patient clusters defined based on K‐means clustering of comorbidities for Long Covid patients between January 2020 and September 2023. Figure S5: Sankey diagram showing the assignment of patients to disease clusters in the pre‐pandemic and post‐pandemic periods. Colours of edges represent the pre‐pandemic cluster to which a patient was assigned. Numbers represent the total patients assigned to each cluster. [file HEX-28-e70429-s001.docx]

**Supplementary results**

#### Comorbidity clusters of Long COVID patients before and after the 1^st^ January 2020

K-means clustering was performed to identify Long COVID patients with similar profiles of pre-existing clinical comorbidities. 1,449 patients did not have a recorded clinical comorbidity prior to 1st January 2020 and were excluded, leaving 2474 patients included in the analysis. The elbow plot in **Figure S1** identified a configuration consisting of 6 clusters to be optimal after 100 repeated initialisations to find the optimal initialisation values for each cluster centroid based on minimisation of the sum of squared distances from the centroid. **Figure S2** shows the prevalence of each clinical comorbidity within each of the 6 clusters identified. The same process was repeated for conditions recorded on or after 1st January 2020. The elbow plot in **Figure S3** identifies a configuration consisting of 9 clusters to be optimal and the frequency of each condition within a cluster is shown in **Figure S4**.

In the pre-pandemic period, four of six clusters are largely defined by the presence of a single comorbidity, namely anxiety, type-2 diabetes, hypertension and asthma (**Figure S2**). A further cluster represents the co-existence of anxiety and depression, while cluster 4 is a mixed cluster, with several conditions of low prevalence, with eczema being the most common. In the post-pandemic clustering of patients, clusters that are qualitatively similar to each of the clusters found in the pre-pandemic clustering are present, with three additional clusters identified (**Figure S4**). Two of these clusters are characterised by the coexistence of two conditions, specifically COPD and chronic lung disease (cluster 2) and type-2 diabetes and hypertension (cluster 6), while a cluster dominated by depressive disorder also appears. **Figure S5** shows patients move between a wide range of clusters from the pre-pandemic to post-pandemic periods. This indicates that the further comorbidities patients with Long COVID may develop are varied and do not follow a narrow set of clinical trajectories.


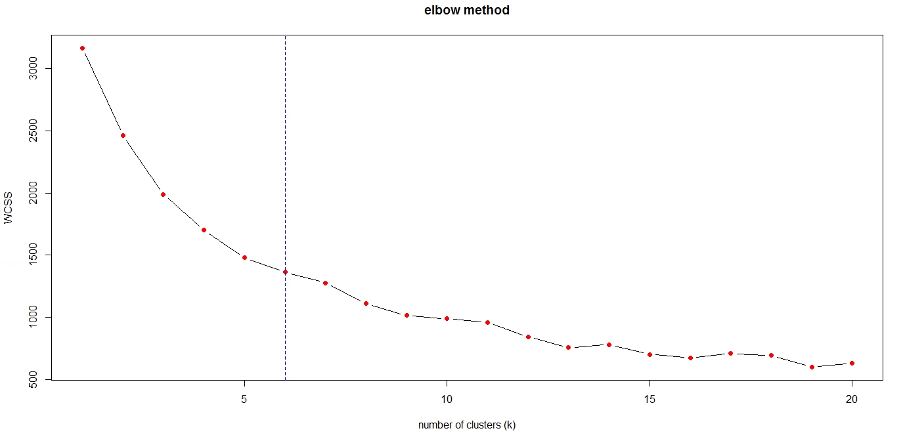


***Figure S1*** *- Elbow plot for K-means clustering of comorbidities for Long Covid patients between January 2017 and December 2019, vertical line indicates the plot chosen configuration of 6 clusters.*


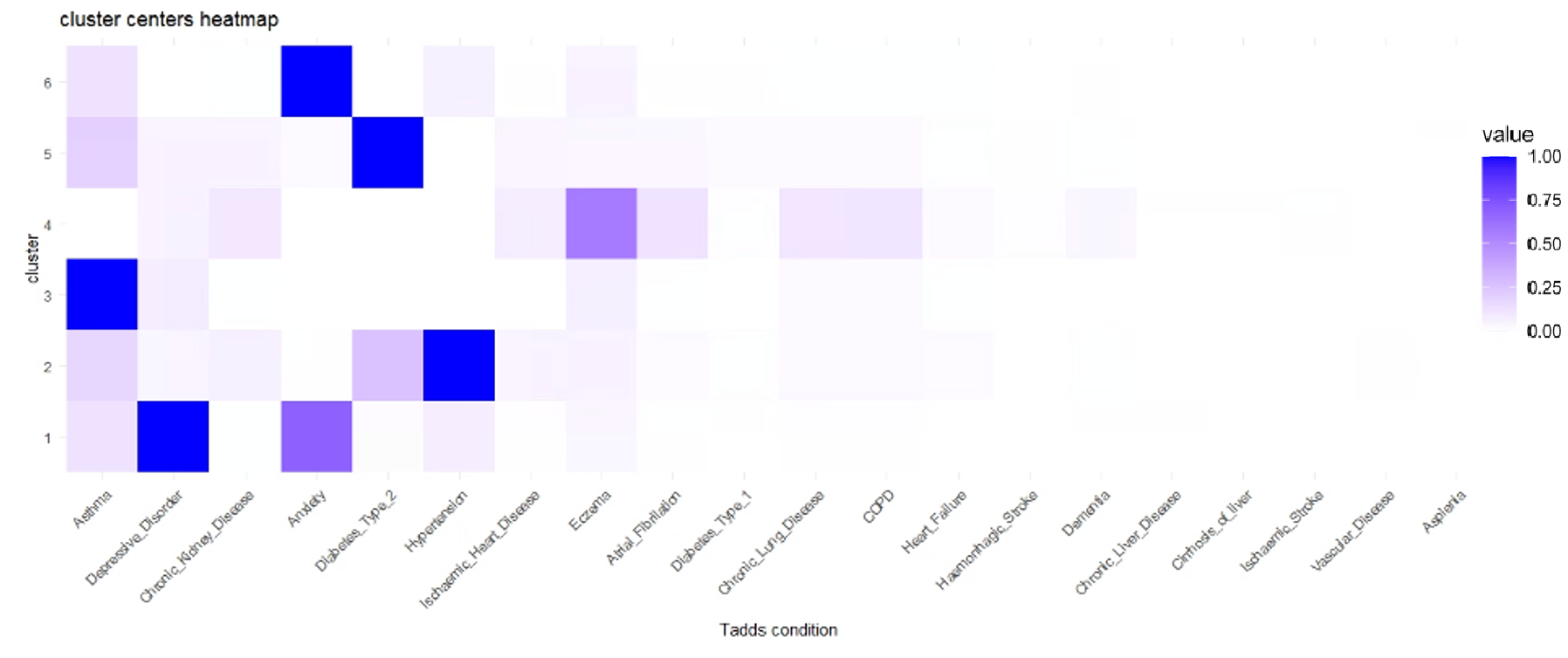


***Figure S2*** *- Heatmap showing the prevalence of conditions within each of the 6 patient clusters defined based on K-means clustering of comorbidities for Long Covid patients between January 2017 and December 2019.*


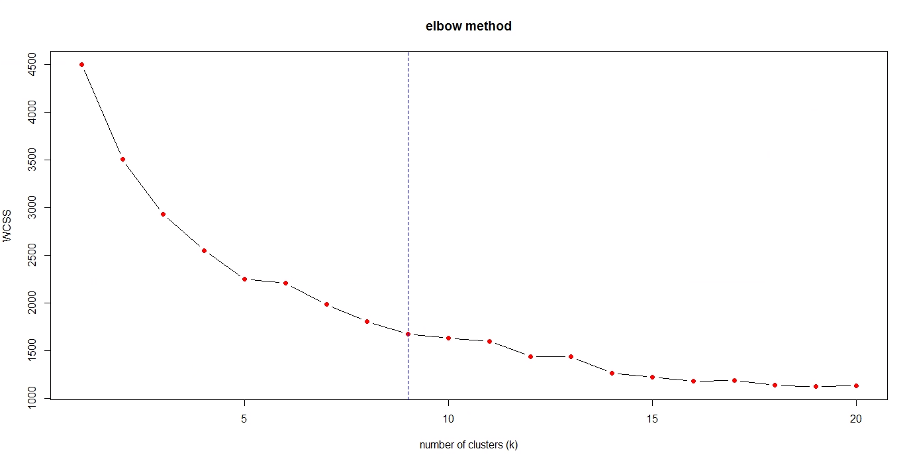


***Figure S3*** *- Elbow plot for K-means clustering of comorbidities for Long Covid patients between January 2020 and September 2023, vertical line indicates the plot chosen configuration of 9 clusters.*


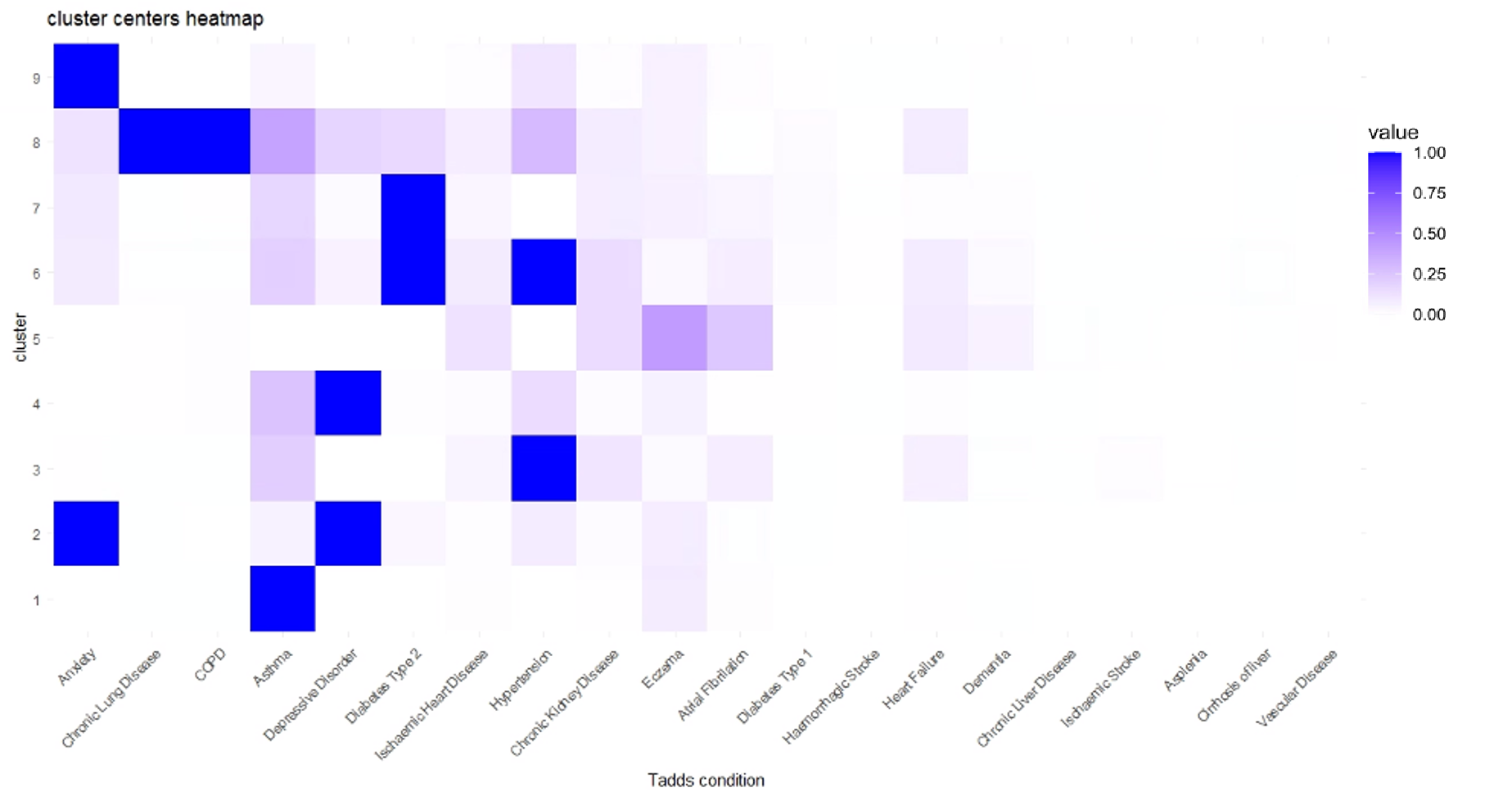


***Figure S4*** *- Heatmap showing the prevalence of conditions within each of the 9 patient clusters defined based on K-means clustering of comorbidities for Long Covid patients between January 2020 and September 2023.*

*
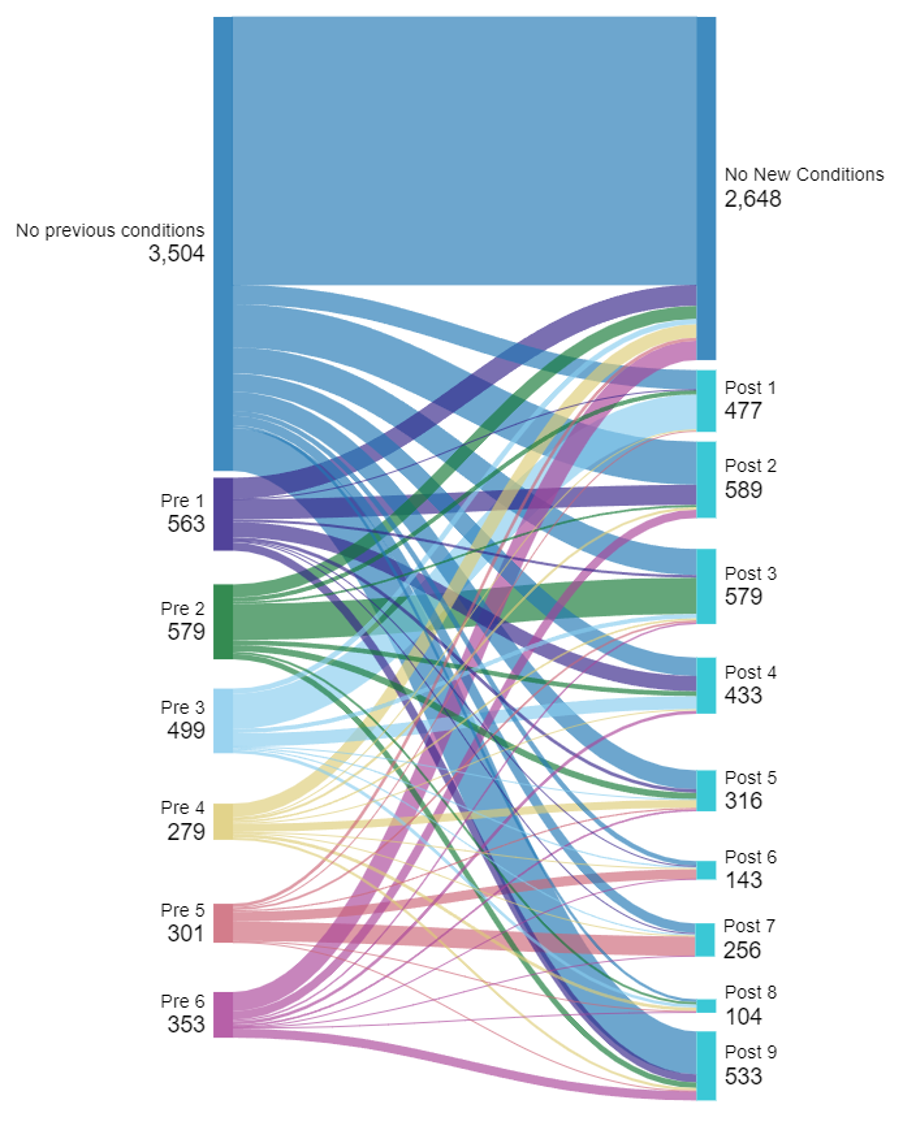
*

***Figure S5*** *– Sankey diagram showing the assignment of patients to disease clusters in the pre-pandemic and post-pandemic periods. Colours of edges represent the pre-pandemic cluster to which a patient was assigned. Numbers represent the total patients assigned to each cluster.*
